# Bacterial Diversity and Community Composition Distribution in Cold-Desert Habitats of Qinghai–Tibet Plateau, China

**DOI:** 10.3390/microorganisms9020262

**Published:** 2021-01-27

**Authors:** Wei Zhang, Ali Bahadur, Wasim Sajjad, Gaosen Zhang, Fahad Nasir, Binglin Zhang, Xiukun Wu, Guangxiu Liu, Tuo Chen

**Affiliations:** 1 Key Laboratory of Desert and Desertification, Northwest Institute of Eco-Environment and Resources, Chinese Academy of Sciences, Lanzhou 730000, China; ziaoshen@163.com (W.Z.); jungleson@163.com (G.Z.); wxk163mail@163.com (X.W.); 2 Key Laboratory of Extreme Environmental Microbial Resources and Engineering, Lanzhou 730000, China; ali_botanist@yahoo.com (A.B.); brightzbl@126.com (B.Z.); 3 State Key Laboratory of Cryospheric Science, Northwest Institute of Eco-Environment and Resources, Chinese Academy of Sciences, Lanzhou 730000, China; wasim.sajjad71@yahoo.com; 4 Key Laboratory of Mollisols Agroecology, Northeast Institute of Geography and Agroecology, Chinese Academy of Sciences, Changchun 130102, China; fa0100@nenu.edu.cn

**Keywords:** cold-desert, proteobacteria, Illumina MiSeq sequencing, geochemical factors, indicator taxa, human activity interference

## Abstract

Bacterial communities in cold-desert habitats play an important ecological role. However, the variation in bacterial diversity and community composition of the cold-desert ecosystem in Qinghai–Tibet Plateau remains unknown. To fill this scientific gape, Illumina MiSeq sequencing was performed on 15 soil samples collected from different cold-desert habitats, including human-disturbed, vegetation coverage, desert land, and sand dune. The abundance-based coverage estimator, Shannon, and Chao indices showed that the bacterial diversity and abundance of the cold-desert were high. A significant variation reported in the bacterial diversity and community composition across the study area. Proteobacteria accounted for the largest proportion (12.4–55.7%) of all sequences, followed by Actinobacteria (9.2–39.7%), Bacteroidetes (1.8–21.5%), and Chloroflexi (2.7–12.6%). Furthermore, unclassified genera dominated in human-disturbed habitats. The community profiles of GeErMu, HongLiangHe, and CuoNaHu sites were different and metagenomic biomarkers were higher (22) in CuoNaHu sites. Among the soil physicochemical variables, the total nitrogen and electric conductivity significantly influenced the bacterial community structure. In conclusion, this study provides information regarding variation in diversity and composition of bacterial communities and elucidates the association between bacterial community structures and soil physicochemical variables in cold-desert habitats of Qinghai–Tibet Plateau.

## 1. Introduction

The cold-desert ecosystem in the northeastern Qinghai–Tibet Plateau is geographically and ecologically distinct. Despite considered an extreme region compared to the surrounding areas, little is known about its microbial diversity and community composition. In addition to extreme fluctuations in annual temperature, other environmental factors, such as low nutrient availability, low-soil-moisture content, and elevated ultraviolet (UV) radiation make the area extremely arid [1,2,3,4,5]. Cold-desert habitats are proposed to reduce the potential productivity of soil or cause destruction of soil system functioning and support relatively simple ecosystems, including food-web structures comprised of cold-adapted plants and microbial taxa [6]. Soil microbes might be the most abundant and diverse types of organisms on Earth [7,8]. Among others, soil microbial diversity and community functions are greatly affected by complex environmental variations [4,9].

Besides, several studies have shown that anthropogenic activities, especially chemical contamination into the desert ecosystems, affect the diversity and richness of soil microbial communities as well as their physiological activities and functional potential of soil microbes [10,11]. Soil microbial communities might be variant owing to increased anthropogenic activities that occur in the railway track, where herbicide is regularly used to control weed growth for the safety of railway tracks [12]. A better understanding of microbial diversity and community dynamics in cold habitats could help to gain insights into the response and adaptation of bacterial communities to extreme environmental conditions. This evidence is vital to evaluate how natural and anthropogenic drivers affect the plant-microbe-soil system under the physicochemical harsh environment.

Soil microbes play a vital role in nutrient availability and maintaining soil quality. Bacteria constitute a key part of the soil biodiversity and play a crucial role in upholding soil processes, which is critical to sustaining the functioning of terrestrial ecosystems [13,14]. Therefore, it is necessary to study how soil bacterial communities are affected by specific environmental changes or disturbances. Previous work suggested that some bacterial community distribution has been well-defined on a large-scale [15,16]. Moreover, additional studies have suggested that spatial distance mainly influences the distribution of bacterial communities. For instance, over large spatial scales, soil pH is the key factor that shapes the bacterial community composition [17,18,19]. In addition, Zhang et al. [20] reported that salinity might be one of the key determinants of bacterial community differences in desert ecosystems. Similarly, bacterial community structure can be influenced by soil temperature [21] and water content (WC) [22,23,24]. Furthermore, compared with soil’s physicochemical properties, microorganisms are often sensitive to fluctuations in the immediate environment and consequently may serve as sensitive indicators of soil health [25,26]. Several microbial indicators have been acknowledged to predict community responses to local environmental habitats. From this perspective, the local scale pattern of soil bacterial communities in different habitats of the cold- desert region of the Qinghai–Tibet Plateau has not been studied until now. In the second stage of the Qinghai–Tibet Plateau railway project from Germu (Golmud) to Lasa, the railway track is 1142 km long. The region contains severe desertification sites, therefore, it was expected that the substantial variation (physiological characteristics, vegetation, and anthropogenic activities) in the cold-desert region.

We hypothesized that the diverse vegetation and anthropogenic activities in the region could drive substantial changes in bacterial diversity and community compositions. To test this hypothesis, three objectives were addressed in the current study: (i) to study the bacterial diversity and community composition of the soil samples collected at a depth of 0–10 cm; (ii) to identify key environmental driving factors and understand their association with bacterial community composition, and (iii) to assess the impact of human activities and vegetation coverage on bacterial community interaction networks.

## 2. Materials and Methods

### 2.1. Study Site Description

The study sites were selected from a cold-desert region in the northwest of XiNing and LaSa, located on the eastern edge of the Qinghai Tibetan Plateau, China, with an elevation of about 2600–3000 m (average elevation of 2800 m). It is a continental desert feature, with a dry and windy climate and flat terrain, a unique alpine, and arid desert landscape. At present, the sandstorm and desertification disasters in the study area are very serious, which restricts the economic and social development of the region. The mean annual temperature is 2.1 °C, with the highest and lowest monthly mean temperature of ~9 °C in June and −5 °C in January. The mean annual precipitation is 374 mm. The main soil type in the study area is sierozem, which is very susceptible to wind erosion.

### 2.2. Experimental Design and Sampling Procedure

**Figure 1:**
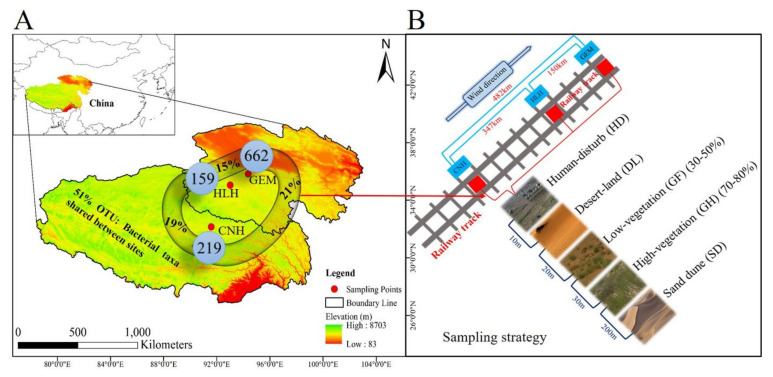
Site biogeography in the bacterial community: (**A**) Map of the Qinghai Tibetan Plateau, China. Geographical distribution of 3 sites where bacterial diversity samples and soil data were collected (Table S1) and showing the operational taxonomic units (OTUs) found in each site (circles) and the percentages shared between different sites (lines) (Figure S2). Shared-OTUs estimates for site pairs are shown. (**B**) Sampling strategy showing the individual soil sample collected (Table S1). CNH—CuoNaHu, HLH—HongLiangHe, GEM—GeErMu.

The Qinghai–Tibet railway project (second phase) from Golmud to Lasa is 1142 km long. The region contains severe desertification sites; therefore, it was expected that the substantial variation (physiological characteristics, vegetation, and anthropogenic activities) in the cold-desert region and hold distinct soil bacterial community composition. Therefore, three sampling sites GeErMu (GEM), HongLiangHe (HLH), and CuoNaHu (CNH) were selected according to anthropogenic activities, vegetation, and desert coverage. Five different habitats were targeted at a specific distance from the railway track as an origin of sampling to ensure a higher level of anthropogenic interference (Figure 1B). Samples were collected from the railway’s track to the sand dune along such transect that had a decreasing trend of anthropogenic activities. Samples were categorized based on habitats: high-vegetation (GH) (70–80%), low-vegetation (GF) (30–50%), desert-land (DL), human-disturb (HD), and sand dune (SD), respectively (Table S1 and Figure 1). In addition, daily based meteorological data were downloaded from the website of China Meteorological Data Service Center (CMDC), i.e., http://data.cma.cn. The meteorological data used in the current study is the average of 30 years (1981–2010) (Figure S1). The dominant vegetation in each site includes (GEM) *Potentilla fruticose*, *Ptilagrostis dichotoma*, *Artemisia demissa*, Carex sp., (HLH) *Leymus secalinus*, *Calamagrostis epigeio*, *Leymus secalinus*, *Saussure* sp., *Stipa* sp., (CNH) *Hippophae thibetana*, *Stipa* sp., *Saussure* sp., *Potentilla bifurca*, *Stipa* sp., *Hippophae thibetana*, *Stipa* sp., *Potentilla bifurca*, *Saussure* sp., and *Tripolium* sp.

In June 2015, at each sampling point, approximately 500 g of soil samples were collected at depth of 0–10 cm. One core was collected from the center of the quadrat (10 m × 10 m) and two soil cores were collected from two corners 8 cm away from the end site of the quadrat and mixed thoroughly. The composite soil was placed in sterile plastic bags and immediately placed in a dry ice box for transportation to the laboratory. The composite soil samples were sieved through a 2.0 mm to remove large gravel. Moreover, the soil samples were air-dried for 1 week and subsequently used for physicochemical characterization, whereas those for bacterial community analysis were immediately stored at −80 °C for later DNA extraction.

### 2.3. Soil Physicochemical Analysis

The soil water content (WC) was determined gravimetrically after drying in an oven at 105 °C for 24 h. Soil pH values were measured after making 1:5 soil: water slurry with a pH meter (Göttingen, PT-10, Sartorius, Germany), and the electric conductivity (EC) of the soil was analyzed using a conductivity meter. Total nitrogen (TN) and total organic carbon (TOC) of soil samples were quantified using an automatic element analyzer (Elementar Vario-EL, Langenselbold, Germany) [27]. The total dissolved solids (TDS) were measured by a conventional conductivity meter.

### 2.4. DNA Extraction, PCR Amplification, and Illumina MiSeq Sequencing

The total DNA was extracted from 0.5 g of each soil sample using the PowerSoil DNA Isolation Kit (MoBio Mo Bio Laboratories, Inc., Carlsbad, CA, USA), following the manufacturer’s instructions. To determine the soil bacterial community diversity and composition in each soil sample, the V4–V5 hyper-variable region of bacterial 16S rRNA gene were amplified using the bacterial universal primer set 515F/806R with the barcode [28]. Quantitative PCR reactions were analyzed using the SYBR^®^ Premix EX TaqTM Ⅱ kit (Takara-Bio Inc., Mountain View, CA, USA), carried out in 20-μL reactions with 10-μL ddH2O, 0.5-μL (10-μM) forward and reverse primers each, 7.0-μL master mix, and 2.0-μL (2.5 ng,1-μL) of template DNA. The amplification of the 16S rRNA genes consisted of initial denaturation at 95 °C for 3 min, followed by 25 cycles of denaturation at 95 °C for 30 s, annealing of 30 s at 50 °C, and an elongation at 72 °C for 30 s, and final elongation step at 72 °C for 10 min.

### 2.5. Bioinformatics Analysis

Raw FASTQ files were cleaned by removing the adapter and used QIIME (1.7) to performed quality-filtered according to the following criteria: (i) the 300-bp reads were truncated at any site that achieved an average quality score of <20 over a 10-bp sliding window, and the truncated files shorter than 50-bp were removed; (ii) exact barcode matching, two nucleotide mismatch in primer matching, and reads comprising ambiguous characters were discarded; (iii) reads that could not be assembled were removed, only overlapping sequences longer than 10-bp were assembled allowing to their overlapped sequence. Open reference operational taxonomic unit (OTU) picking was done with pick_open_reference_otus.py by the default uclust method dataset (97% similarity cutoff was used, alpha issue, download from the web site of QIIME http://qiime.org/home_static/dataFiles.html), and singletons were discarded throughout OTU picking. The phylogenetic association of each sequence was analyzed by RDP Classifier (http://rdp.cme.msu.edu/) against the dataset using a confidence threshold of 97%. Single rarefaction.py in QIIME was used to generate OTUs table with even reads in each soil sample.

### 2.6. Statistical Analysis and Data Visualization

Statistical analysis was undertaken by using base R (v.3.6.1; https://www.r-project.org/) specific packages, SPSS, and PAleontological Statistics (PAST) (Version 3.2) software. The figure, excluding maps, were made using ggplot2 packages in R [29].

The alpha diversity indices including the Shannon index, Chao richness, and Simpson index were analyzed to compare the diversity and richness of bacterial communities [30]. A Bray Curtis distance tree (at a ≥97% sequence similarity level) of the bacterial community across the different habitats were calculated using PAST with the statistical computing paired group (UPGMA). The relative abundance of the dominant phyla and genera in each sample on the composition of the bacterial community, simulated abundance matrix, and grouping then standardized abundance, ggplot2 draws a stacked chart. Differences between samples were visualized by Non-Metric Multidimensional scaling (NMDS) analysis based on Bray-Curtis distance (*metaMDS* function, vegan) [31]. Analysis of similarities (ANOSIM) was used to test whether the differences between different groups were significant, using the R package vegan (*anosim*) [32]. Moreover, an NMDS plot was created using the correlation between responsive variables and bacterial taxonomic composition across different habitats. PERMANOVA (999 permutations) was performed to assess the differences in community composition across the sites.

The characterization of bacterial features differentiated across the different sampling sites at different taxonomic levels was performed using the LDA effect size LEfSe method (LDA > 4.0) for biomarker discovery. To reveal the co-occurrence patterns of bacterial communities based on the relative abundances of genera were retained for network construction. The genera and environmental factors data files were organized as guided in the pipeline. All potential pairwise Spearman’s rank correlations between the genera were calculated with “*vegan*” (*psych*) packages in R. A cutoff value (adjusted *p* < 0.05) was incorporated into the network analysis [33]. Network visualization was undertaken with “Gephi” random networks of equal size that were built as actual networks for community comparisons. The Venn diagram was drawn using the “*VennDiagram*” package [34].

## 3. Results

### 3.1. Soil Characteristics of the Sampling Sites

The sampling sites across the different habitats significantly varied in terms of soil physicochemical characteristics (see Table 1). Soil pH was not significantly different in all habitats, ranging from 7.6 to 8.6, which shows a slightly alkaline nature, but higher pH values were recorded in HD across all three sites (GEM, CNH, and HEL). EC in the soils ranged from 26.42 to 89.63 μS/cm, it was significantly higher in G-HD and H-HD than C-HD, a similar trend was found for TDS mg/L. SAL in the soils ranged from 0.02 to 0.06 practical salinity unit (psu) of the soil samples, and it was higher in HD than in other habitats. The highest WC percentage was 11.13, 10.65, and 9.18% for GH. The TN content did not differ across the habitats in either site ranged from 0.03 to 0.06%. The only significant difference was observed in the TN for G-HD. TOC in the soil ranged from 0.31 to 1.06%, it was significantly higher in G-GH and C-HD (Table 1). Generally, the soil characteristics in the G-HD and H-HD were quite different from those in the other habitats.

### 3.2. Bacterial Richness, Diversity, and Taxonomic Composition

The total raw sequencing data were thoroughly processed by mothur and exceeded 384,598 quality-filtered of 16S rRNA sequences across the habitats (five habitats per site). The sequence library of each habitat, after high-quality filtering hold 17,251 to 37,877 sequences (Table 2). The total abundance, 23,303 OTUs were annotated (identity 97%) in the complete data set, varying from 962 to 2219 across the region. The Shannon and Simpson indices were compared, and bacterial diversity was evaluated across the habitats (4.15–6.58 Shannon; 0.004–0.131 Simpson) (Table 2). The range of Chao was 1304 to 2767, whereas the range of ACE was 1347 to 2722, which were considered as richness indices. G-DL had a very high level of the Chao index and ACE, consistently, had the leading number of OTUs. Furthermore, H-GH had a low level of Chao and ACE, and the smallest number of OTUs (Table 2). The OTUs rarefaction curves showed that diversity was completely examined in all samples (Figure S3 and Table 2). Besides, G-DL habitats had the highest bacterial diversity and also had high community richness. In short, our results indicated that by likening the two index values, we found that the richness index was generally positive to the diversity index.

**Figure 2:**
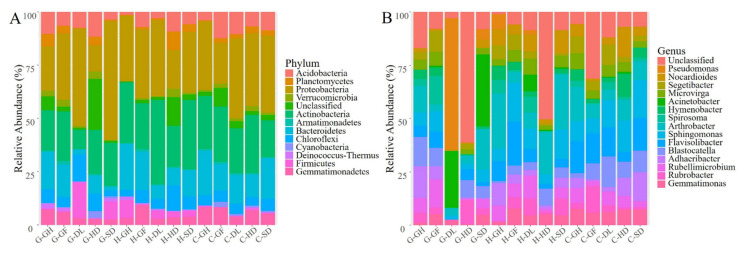
The bacterial composition of different habitats across 3 sites sampled from cold-desert, Qinghai–Tibet Plateau, China: (**A**) Relative abundance of the dominant bacterial phylum, (**B**) Relative abundance of the dominant bacterial genus are shown in stack plot. The relative abundances are based on the ≥97% similarity clusters of the OTUs.

The OTUs were classified into 42 phyla, 99 classes, 219 orders, 420 families, 792 genera. Across all habitats, the explanation for a large proportion of the phyla was as follows, Proteobacteria (12.36–55.72%) was largely dominant phylum, followed by Actinobacteria (9.21–39.69%), Bacteroidetes (1.81–21.54%), and Chloroflexi (2.65–12.63%). Interestingly, unclassified phyla were dominated in HD (23.92% in G-HD to 13.33% in H-HD) soil samples (Figure 2A). At the taxonomic level of genera, the bacterial communities of different habitats across 3 sites were significantly dissimilar (Figure 2B). *Sphingomonas* (1.91–18.10%) and *Arthrobacter* (3.36–25.96%) were the most abundant genera within all habitats. *Flavisolibacter* (20.65%) was the most abundant genus in the H-GF habitat and the second most abundant genus in C-SD (15.433%) habitat. Remarkably, *Pseudomonas* was a dominant genus in G-DL (61.49%) habitat, whereas, only a few *Pseudomonas* OTUs were detected in all other habitats (Figure 2B). *Acinetobacter* was the most abundant genus within the G-SD (33.85%) and G-DL (26.68%). Notably, a major portion of the abundant genera was unclassified in the G-HD (61.21%) and H-HD (50.28%), whereas C-HD displays the diverse abundant trend of genera (Figure 2B). A similar significant difference was detected at the genus level (Figure S4). Taken together, the results encompassed that soil bacterial community composition in the study sites were high, and there is some unique unclassified bacterial community which presents in the human-disturb habitats.

### 3.3. Distribution of Bacterial Community Across the Habitats

**Figure 3:**
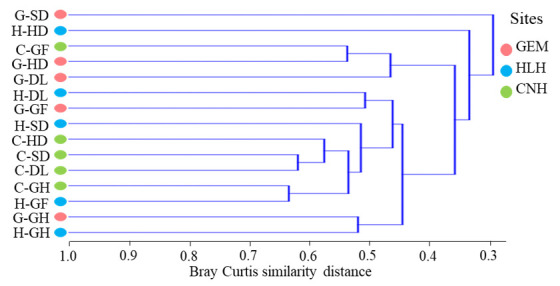
A Bray Curtis distance tree (at a ≥97% sequence similarity level) of the bacterial population of the cold-desert, Qinghai–Tibet Plateau, China: The tree was generated using Algorithm PAST software with the statistical computing paired group (UPGMA). The distance for each pair of samples (habitat) is showed by the position of the node between them, according to the Bray Curtis similarity indices. More details are shown in Table S3. CNH—CuoNaHu, HLH—HongLiangHe, GEM—GeErMu.

OTUs cluster analysis based on the Bray Curtis similarity index paired group process with algorithm mean was estimated (Figure 3). The dendrogram revealed five clustered groups. The members of the first group encompassed only G-SD; the second group (H-HD) clustered alone and exhibited different bacterial communities. The third group included C-GF and G-HD; the fourth group members consisted of H-DL, G-GF, H-SD, C-HD, C-SD, C-DL, C-GH, and H-GF. G-GH and H-GH have consisted of the fifth group (Figure 3). Therefore, it was concluded that the H-HD cluster alone and had different bacterial community composition. A higher similarity was detected in the CNH sampling sites excluding C-HD.

**Figure 4:**
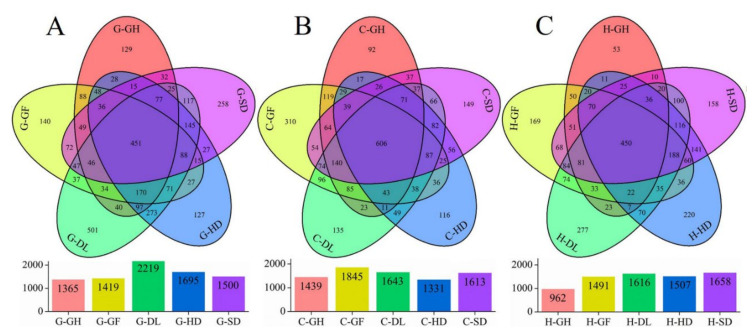
Variation-partitioning analysis for explaining soil bacterial diversity by different habitat across three sites sampled from cold-desert, Qinghai–Tibet Plateau, China: which was shown in a five-way Venn diagram. The shared and unique OTU found in each site and habitat are represented by different circles and clusters per segment. (**A**) GEM site; The number of OTU (451) was shared by all of the bacteria with each site; (**B**) CNH site; shared OTU (606); (**C**) HLH site; shared OTU (450), respectively. CNH—CuoNaHu, HLH—HongLiangHe, GEM—GeErMu.

Venn diagram revealed that the distribution of OTUs was different across the habitats (Figure 4). In total, of the 8198 OTUs, 7871 OTUs, and 7234 OTUs were detected at GEM, CNH, and HEL sites, respectively, only 451, 606, and 450 OTUs were shared across the five habitats, respectively (Figure 4). In the GEM site, the number of OTUs was unique in each habitat such as 501 (G-DL), 258 (G-SD), 129 (G-GH), 127 (G-HD), and 140 (G-GF), accounting for the total observed OTUs (Figure 4A). In the CNH site, the number of unique OTUs was highest at C-GF (310) while the lowest were detected at G-GH (92) (Figure 4B). However, the HLH site recorded the highest number of unique OTUs at H-DL (277) whereas it was lowest at H-GH (53) (Figure 4C). The number of lowest unique OTUs across the habitats was found at C-GH and H-GH. Therefore, the insertion of the other habitats into the G-HD, C-HD, and H-HD seemed to have affected the community structures, and more OTUs were shared across different communities. These results indicated that the distribution of OTUs responded differently to each habitat, and all the cold-desert natural and anthropogenic habitats had significant impacts on the bacterial community distributions.

**Figure 5:**
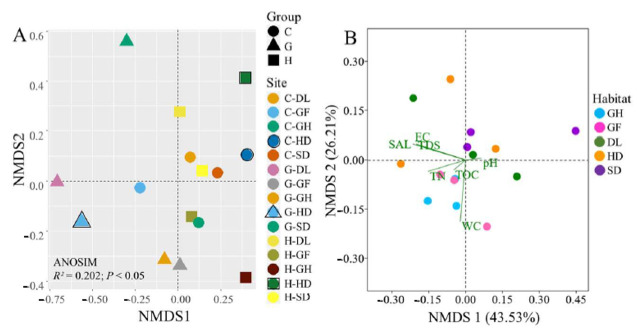
Bacterial communities of different habitats across three sites sampled from cold-desert, Qinghai–Tibet Plateau, China: (**A**) Non-metric multidimensional scaling (NMDS) of the Bray–Curtis-based dissimilarity matrix of bacterial community’s structure. Soil samples are divided into three groups (circle—C; triangle—G; rectangle—H). Analysis of similarities (ANOSIM) metric, a number indicating the degree of differences between the group (R^2^ = 0.202; *p* < 0.05), for comparisons of bacterial communities. (**B**) NMDS showing the correlation between responsive variables (environmental factors) and microbial taxonomic composition (explanatory variables) across different habitats. The green lines show the direction of the maximum change in soil variables (TN—total nitrogen; WC—water content; TOC—total organic carbon; SAL—salinity; TDS—total dissolved solid; EC—electric conductivity). First and second ordination axes were plotted, representing 43.53% and 26.21% of the variability in the data set, respectively. CNH—CuoNaHu, HLH—Hon gLiangHe, GEM—GeErMu.

A comparison of the bacterial community structure for different habitats based on NMDS analysis revealed that G-HD was separated from the other two C-HD and H-HD habitats. Verifying this, permutational multivariate analysis of difference shown significant variances in bacterial community composition across the habitats (ANOSIM metric, a number indicating the degree of differences between the group *R^2^* = 0.202, *p* < 0.05) (Figure 5A).

### 3.4. Identification of Environmental Variables Shaping Cold-Desert Bacterial Community Structures

**Figure 6:**
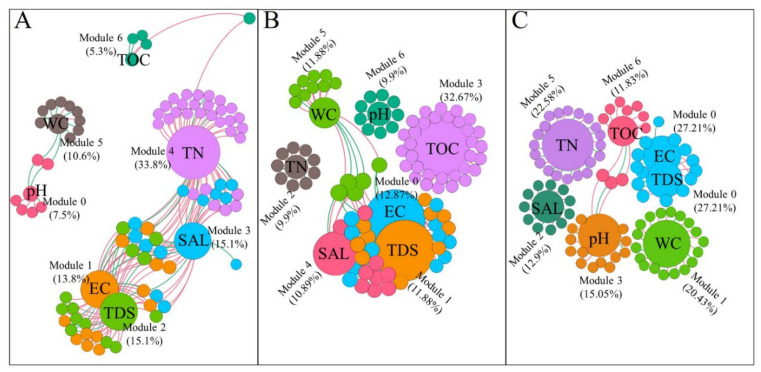
Co-correlations network of the bacterial communities of different habitats across three sites collected from cold-desert, Qinghai–Tibet Plateau, China, based on a correlation analysis at the genus level (relative abundance >0.01%; Spearman’s correlations adjusted *p* < 0.01). (**A**) The samples from site GEM, nodes 94, and edges 164. (**B**) The samples from site HLH, nodes 101, and edges 157. (**C**) The samples from site CNH, nodes 93, and edges 102. Edges characterize significantly positive (green) or negative (red) correlation. Nodes sizes correspond to their relative abundances in the data set. Genera proportions in each module are shown in Table S4. CNH—CuoNaHu, HLH—HongLiangHe, GEM—GeErMu.

To assess the relationship between soil variables and bacterial communities, the constrained analysis of NMDS was performed (Figure 5B). Overall, the first two axes NMDS1 and NMDS2 explained 69.74% of the variation in the bacterial community structure. The partial NMDS indicated that TN, TOC, and EC were the largest factors of the soil (Figure 5B). In Figure 5B, the GH and GF samples relatively plotted closed compared to other samples. Across the selected soil variables, TN (*R^2^* = 0.24, *p* = 0.005) and EC (R^2^ = 0.23, *p* = 0.006) were important soil variables that correlated with the bacterial community composition in the habitats. However, the other soil variables, including pH, WC, TOC, TDS, and SAL were not significantly correlated with bacterial community composition (Table S2). A co-occurrences network analysis was conducted to assess the correlation between the environmental variables and bacterial communities at the genus level across the different sites (GEM, HLH, and CNH) (Figure 6). At the GEM site, the network analysis had 94 nodes linked by 162 edges, at the HLH site, 101 nodes linked by 157 edges, and the lowest linked consist 93 nodes associated with 102 edges at the CNH site (Figure 6A–C; Table S4). The number of negative relationships (77.16, 55.51, and 61.78%) was considerably larger than the number of positive relationships (22.84, 44.49, and 38.24%) at GEM, HLH, and CNH, respectively. The average degree (avg K) value of the CNH site was higher than the values of the other two sites. Moreover, the modularity of the HLH and CNH co-occurrence network was higher than 0.4, indicating a more modular structure compared to GEM (<0.4) (Figure 6A–C; Table S4). Modules 4 accounted for 33.8% of network nodes with TN at the GEM site (Figure 6A) and at the CNH site (modules 5 accounted for 22.58%). Whereas, at site HLH, modules 3 accounted for 32.67% of network nodes with TOC (Figure 6B). The environmental variables in the different habitats were highly diverse, therefore, the results indicated that local environmental factors are vital for shaping bacterial community structure on a spatial small scale.

### 3.5. Indicator Taxa in Soil Bacterial Communities

**Figure 7:**
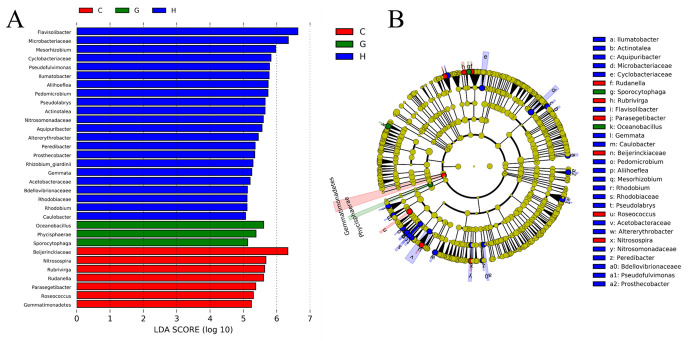
(**A**) Visualization of indicator genera distribution across different habitats in the 3 sites sampled from cold-desert, Qinghai Tibet Plateau, China: (**A**) Indicator bacterial groups within the three sites with linear discriminant analysis (LDA) scores higher than 3. (**B**) Cladogram indicating the phylogenetic distribution of bacterial lineages. The colored nodes from the inner to the outer circles represent taxa from the phylum to genus level. The significantly diverse taxa are signified by different colors representing the three sites. C (CNH—CuoNaHu), H (HLH—HongLiangHe), G (GEM—GeErMu).

To assess the significance of discriminatively specific bacterial taxa, the LEfSe analysis was accomplished from the phylum to genus across the three sites using the default logarithmic (LDA) value of 4 (Figure 7A). The clade graph showed that 22 groups of bacterial taxa were enriched in the HLH site samples, 7 groups in the CNH followed by GEM (3 groups of bacterial taxa), respectively.

The community compositions of the bacterial community between the three sites were significantly different, as displayed in the LEfSe cladogram (Figure 7B). Most of the clades across the sites were unclassified. After removing the unclassified clades, the results showed that different groups at different taxonomic levels can be significantly distinguished across the sites. For instance, *Gemmatimonadetes* acted as a leading discriminant clade at site CNH. *Phycisphaerae* is the most differently abundant bacterial class at GEM. At the family level, *Cyclobacteriaceae*, *Microbacteriaceae*, *Bdellovibrionaceae*, *Nitrosomonadaceae*, *Acetobacteraceae*, and *Rhodobiaceae* were significantly enriched at the HLH site, whereas *Beijerinckiaceae* was dominated at CNH (Figure 7B).

## 4. Discussion

Deserts contribute disproportionately to the terrestrial biodiversity of Earth, especially in the cold-desert soil, where they host hotspots of variation microbial diversity. With about 109–1010 bacterial cells per gram of soil, bacterial diversity varies markedly across the desert ecosystem [35]. Altogether, over the past decade, studies have revealed that certain bacterial taxa are distributed in hostile environmental conditions such as cold-desert. These bacteria adapt special protective strategies to cope with environmental stresses [36]. Comparatively very few reports are present on the bacterial diversity in different habitats across the Qinghai–Tibet Plateau desert. Most of them have focused on the bacterial communities of gas fields, salts, and/or contaminated lakes [37,38]. This study presents a comprehensive assessment of bacterial diversity and structural analyses in different habitats across the Qinghai–Tibet Plateau. Significant variations were reported in physicochemical parameters in the study area. Nevertheless, the harsh characteristics of cold-desert regions vary fundamentally from those of green mountainous regions and are expected to play a key role in maintaining and generating bacterial diversity. With continuous human interference through railway tracks and land use, the role of the desert as refugia for bacterial diversity may come under risk. Our findings consequently demonstrate that different habitats in cold-desert ecosystems harbor significantly diverse bacterial communities.

### 4.1. Variation in Bacterial Diversity and Community Composition

Our findings highlight that bacterial communities had a noticeable difference across the desert habitats (Figure 5), and that bacterial richness and abundance had a dramatic variation (Table 2). Similar findings have been reported for other dry-land desert ecosystems, showing a strong effect of different habitats aspects on bacterial communities, including karst rocky desert [39] and high arctic desert [40]. In this study, bacterial abundance and richness were higher than those in previous studies in the Badain Jaran desert and Tenger desert [41], and cold desert ecosystem [42], this might be attributed to the methodological variances and region selections. In our study, human-activities and vegetation habitats might affect soil bacterial diversity. Similar findings have been reported in previous studies such as Kuwait desert soils [43] and Drass, cold-desert in western Himalaya [44]. Unexpectedly, these findings suggest that the different habitats from cold-desert in the Qinghai Tibet Plateau comprise more bacterial diversity and the desert environment consists of bacteria having a large reservoir of genetic variability. Since we found 451, 606, and 450 OTUs in common across the five habitats (GEM, CNH, and HLH sites), the five habitats likely comprise different OTUs (Figure 4A–C), as previously reported in desert ecosystems [45,46]. The variances in bacterial composition across the habitats were significant at the OTUs level, even for the two close locations (HLH—4633 elevation and CNH—4624 elevations). Our results provide evidence that the desert environment comprises numerous different OTUs depends on the habitats rather than the geographical locations.

The bacterial communities were attributed to different phyla, and 12 phyla dominated all samples. This level of diversity recommends a larger ecosystem complexity in the cold-desert habitats than previously appreciated [41,42] (Figure 2A). The bacterial diversity in this study was dominated by desiccation and radiation-resistant bacteria that were reported in previous studies [4,26,43]. The relatively low number of bacterial communities and taxonomic evenness, coupled with the presence of unclassified phyla, show that the HD habitat may characterize a transient soil-borne inoculum rather than the indigenous community. In contrast, the specific existence of Chloroflexi across the habitats, the phylum acknowledged to be important in tundra soils [47] recommends a true soil community might occur. The occurrence of Firmicutes in other cold habitats is well reported in the literature [48]. Previous reports on Himalaya regions, Pradhan et al. [49] and Shivaji et al. [50] stated that Firmicutes as the dominant phylum contrary to our study. Cold-deserts are representative of physicochemical extreme environments, where the scarcity of water is the basic parameter affecting the bacterial community. Therefore, xerophilic bacterial communities that are adapted to comparatively high radiation and low temperatures are likely to be the leading communities in these ecosystems.

The presences of unclassified genera were surprising that indicate the anthropogenic influence on bacterial diversity and the site is rich in novel phylogenetic taxon (Figure 2B). It has been previously stated that anthropogenic activities are significant sources of contamination [43,51,52]. Recently, the identification of unclassified taxa has motivated a new wave in research. In particular, to elucidate and quantify their contribution to biological processes and influences of essential ecological variations [4,20,26]. Conversely, the specific occurrences of rhizospheric bacteria in vegetative habitats can be attributed to their role in plant promotion in Qinghai Tibet Plateau compared to other desert habitats that lack the existence of rhizospheric bacteria [43,53].

Across the region, the samples (except for C-GF) in CNH are closely grouped compared to the samples in sites GEM and HLH. This grouping shows that the bacterial community structure of cold-desert habitats in a different location has spatial heterogeneity, which is consistent with previous studies [14,26,54]. Interestingly, the samples from GEM and HLH plotted far away and showed higher dissimilarity. Though the study area was narrow, yet the spatial separation, environmental characteristics, and/or species-sorting mechanism [55] are the key defining variables that influenced the bacterial community’s composition.

### 4.2. Bacterial Communities in Relation to Environmental Variables

It has been previously reported that environmental factors such as WC, TOC, TN, salinity, and pH are vital in shaping the bacterial community in cold-deserts [44,56,57]. In the present study, environmental variables significantly explained the variation of bacterial communities across the region. Here, we detected that TOC, TN, and EC content were relatively the key variables in desert soil samples (Table 1), some of these variables have earlier been implicated in correlations with bacterial diversity [26,58]. In this study, pH was significantly positively correlated with Actinobacteria and our results are in line with a previous study [59]. In the previous studies, the bacterial community composition was most affected by TN and TC contents and soil physical variables like WC, but not pH [60], while Ca, C, K, and WC influenced the structure and distribution of microbial communities in the Antarctic valley [56]. Actinobacteria are adjusted to oligotrophic environments where the hyphae allow the bacteria to restore nutrients and moisture through pores in the soil [20,61]. Cyanobacteria are positively correlated with soil variables and play an important role in the N and C economy of desert soil [62]. Of note, SAL has earlier been recommended as an important driver of bacterial community diversity [63,64]. Furthermore, the significant influence of environmental factors was reported on overall OTUs abundance in NMDS analysis. The environmental factors in the study sites differ greatly, particularly TN, TOC, and EC, which recommends that local environmental factors are the key determinants that can determine the bacterial diversity on a small special scale (Figure 5B), as previously reported in the western Tibetan Plateau [26,65]. Samples GH and GF showed relatively higher similarity that might be due to the common ecological setup provided by vegetation coverage. Similarly, the diverse bacterial community can consume soil nutrients and drive biogeochemical cycling and launch a constant ecosystem in the local niche.

### 4.3. Network Interaction Differences and Potential Indicators

The responses of different network modules were distinct in different sites (Figure 6). TN showed the maximum number of associations with the bacterial community in GEM and CNH sites while TOC showed maximum interaction in the HLH site. Likewise, TOC might support the predominant genera *Arthrobacter* in the dune habitat (HLH site) (Figure 2 and Figure S5). This finding might be supported by the Jiang and Yang [66] study, which showed that the north of the desert contains wide oases and organic carbon carried by dust to the desert. Overall, these findings strongly suggest that habitat-filtration may serve as an influential practice defining dune bacterial community assemblies [67]. This result chains the notion that bacterial community assembly is governed by environmentally driven functional features [18] and soil nutrients are crucial in this regard. Regarding the interspecific interactions, we found stronger negative relations than positive between environmental variables and bacterial communities across the study sites. For instance, positive relationships were found between the Actinobacteria and Proteobacteria (Figure S4) and they are both chemoheterotrophic and aerobic bacteria [68]. Furthermore, different genera of Firmicutes and Actinobacteria can induce dry cold habitats producing endospores or forming spores, respectively; they can also produce antimicrobial substances affecting an extensive spectrum of microorganisms [69]. On the contrary, Fierer et al. [70], reported an increased abundance of antimicrobial-resistance genes in non-desert habitats than desert habitats. This finding signifies that competitive interactions are not as vital in shaping desert bacterial communities. In the notion of these conflicting statements, more emphasis on species interactions, the relative assistances of stochastic and deterministic processes, and how these differ via time and with environmental variables, is required.

Taxa within bacterial communities that quickly respond to habitat variation are considered as potential indicators [71]. Due to local ecological variation, bacterial taxa sensitive to the environmental changes in each site and habitat (Figure 7). Soils from the HLH site indicating that the number of significant differences in bacterial taxa was higher than that of GEM and CNH sites. Some bacteria are retrieved from the vegetative habitat of the HLH site, such as *Rhizobium* and *Sphingomonas*, the respective bacteria were also revealed in the plant rhizosphere of Taklimakan desert southern edge [52]. The phylum Gemmatimonadetes are found in a CNH site, which is distributed in a wide range of habitats and contains oligotrophic bacteria with good adaptation to low soil moisture content [72]. In this framework, it is worth stating that the soil moisture content was much lower in the desert and sand dune land (~4–5%, except H-DL habitat) than that in vegetation habitats (~5–11%). The presence of thermophilic bacteria, such as *Spirosoma*, *Adhaeribacter*, and *Rubellimicrobium* might be related to the formation of soil crust [73]. Excitingly, Deinococcus–Thermus is an acknowledged group of extremophilic bacteria that could endure in ionizing radiation environments [74], but in the current study their abundance was not sophisticated. These distinctive potential indicators deliver evidence to well respond to environmental variation occurring in the cold-desert habitats, which is in line with other studies conducted in cold environments [75].

## 5. Conclusions

Regardless of the cold and dry nature of the Qinghai–Tibet Plateau desert soils, this study found a relatively high diversity within bacterial community composition. Moreover, this study provides the first insight into bacterial communities associated with human-disturbed, vegetation coverage, desert land, and sand dune habitats across the desert soil. Higher variations in the bacterial communities among the cold-desert desert sites suggest the site-specific variation of different habitats. The bacterial community structure could be best described by TN and EC levels in the soil and much different from other soil physiochemical variables that exist in extremely cold conditions. As a whole, this work provides a comprehensive study that should be used as a foundation for future studies, likewise, unclassified genera were dominated in human-disturbed habitats, for such aspect, many unknown bacterial species appeared to be present and remain to be identified and characterized in future studies.

## Figures and Tables

**Table 1:** Soil properties (mean ± SE) of different habitats across 3 sites soil sampled from cold-desert, Qinghai–Tibet Plateau, China. Different letters indicate significant differences across the habitats for each site based One-way ANOVA and Duncan comparisons. *F* and *p*-values obtained in ANOVA’s are shown at the bottom of each site. CNH—CuoNaHu, HLH—HongLiangHe, GEM—GeErMu.

Site	pH	EC (μS/cm)	TDS (mg/L)	SAL (psu)	WC (%)	TN (%)	TOC (%)
G-GH	7.88 ± 0.03a	61.82 ± 1.50c	31.19 ± 0.46c	0.04 ± 0.01b	9.18 ± 1.04b	0.04 ± 0.00a	1.05 ± 0.13b
G-GF	7.81 ± 0.09a	46.18 ± 0.69b	23.31 ± 0.46b	0.03 ± 0.00b	6.74 ± 1.05ab	0.03 ± 0.00a	0.38 ± 0.02a
G-DL	7.92 ± 0.07a	45.42 ± 0.25b	22.68 ± 0.07b	0.03 ± 0.00b	5.58 ± 0.40ab	0.04 ± 0.00a	0.49 ± 0.02a
G-HD	8.29 ± 0.28a	89.63 ± 0.76d	45.12 ± 0.27d	0.06 ± 0.01c	4.42 ± 0.86a	0.06 ± 0.00b	0.50 ± 0.01a
G-SD	7.84 ± 0.01a	36.31 ± 0.88a	17.79 ± 0.66a	0.02 ± 0.01a	4.87 ± 0.32ab	0.03 ± 0.00a	0.48 ± 0.08a
*F*	2.00	534.45	616.12	13.83	5.92	17.28	14.71
*p*	>0.05	0.000	0.000	0.007	0.039	0.004	0.006
H-GH	7.62 ± 0.11a	33.29 ± 0.86b	16.73 ± 0.41b	0.02 ± 0.00a	10.65 ± 0.50c	0.04 ± 0.00a	0.38 ± 0.04a
H-GF	8.33 ± 0.32a	37.31 ± 0.88b	17.92 ± 0.79b	0.03 ± 0.00ab	5.18 ± 0.89b	0.04 ± 0.01a	0.36 ± 0.01a
H-DL	8.30 ± 0.25a	26.42 ± 0.86a	13.20 ± 0.55a	0.02 ± 0.00a	10.28 ± 0.45c	0.04 ± 0.01a	0.31 ± 0.06a
H-HD	8.57 ± 0.39a	64.87 ± 0.90d	32.42 ± 0.30d	0.04 ± 0.01b	4.29 ± 1.29ab	0.04 ± 0.00a	0.39 ± 0.04a
H-SD	7.98 ± 0.01a	44.15 ± 1.83c	22.84 ± 1.08c	0.04 ± 0.01ab	1.86 ± 0.65a	0.04 ± 0.00a	0.34 ± 0.00a
*F*	1.97	168.88	118.24	3.20	22.35	0.53	0.71
*p*	>0.05	0.000	0.000	0.177	0.002	0.720	0.62
C-GH	7.73 ± 0.07a	45.71 ± 2.17ab	23.32 ± 0.78ab	0.03 ± 0.01a	11.13 ± 2.39c	0.05 ± 0.00a	0.72 ± 0.03a
C-GF	7.76 ± 0.01ab	52.45 ± 2.99c	26.15 ± 1.50b	0.04 ± 0.01a	9.24 ± 0.11bc	0.05 ± 0.00a	0.99 ± 0.08b
C-DL	7.71 ± 0.03a	41.39 ± 1.37a	20.89 ± 0.87a	0.03 ± 0.01a	4.72 ± 1.18ab	0.05 ± 0.01a	0.86 ± 0.04ab
C-HD	7.96 ± 0.04b	42.37 ± 0.18ab	21.26 ± 0.18a	0.04 ± 0.01a	4.88 ± 0.31ab	0.04 ± 0.00a	1.06 ± 0.01b
C-SD	7.92 ± 0.01ab	48.87 ± 0.79bc	24.13 ± 0.52ab	0.03 ± 0.02a	4.33 ± 0.93a	0.05 ± 0.00a	0.96 ± 0.08b
*F*	8.96	6.53	5.98	0.46	5.99	0.55	5.69
*p*	0.017	0.032	0.038	>0.05	0.038	0.709	0.42

**Table 2:** Richness and diversity estimates for Illumina libraries from different habitats across 3 sites of soil sampled of cold-desert, Qinghai Tibet Plateau, China. CNH—CuoNaHu, HLH—HongLiangHe, GEM—GeErMu.

Site	Reads	OTU	Ace	Coverage	Chao	Shannon	Simpson
G-GH	19,564	1365	1834	0.98	1823	5.67	0.012
G-GF	35,322	1419	1707	0.99	1739	5.64	0.011
G-DL	22,929	2219	2722	0.97	2767	6.58	0.004
G-HD	23,445	1695	2109	0.98	2066	5.74	0.017
G-SD	37,877	1500	1722	0.99	1715	4.15	0.131
H-GH	17,251	962	1347	0.98	1304	5.08	0.021
H-GF	24,239	1491	1885	0.98	1914	5.98	0.006
H-DL	30,447	1616	1957	0.99	1924	5.41	0.023
H-HD	21,065	1507	1928	0.98	1976	5.84	0.010
H-SD	29,558	1658	2171	0.98	2188	5.73	0.013
C-GH	19,755	1439	1906	0.98	1975	5.87	0.008
C-GF	31,367	1845	2220	0.99	2243	5.95	0.010
C-DL	25,934	1643	2172	0.98	2144	5.86	0.008
C-HD	19,231	1331	1731	0.98	1732	5.68	0.012
C-SD	26,614	1613	2113	0.98	2079	5.80	0.009

## Data Availability

The data presented in this study are available on request from the corresponding author.

## Supplementary Materials

The following are available online at https://www.mdpi.com/2076-2607/9/2/262/s1, Figure S1: (A) Temperature data obtained from ordinary Inverse distance weighting (IDW) interpolation based on the 30-year average annual records (1981 to 2015). (B) Precipitation map obtained from ordinary Inverse distance weighting (IDW) interpolation based on the 30-year average annual records (1981 to 2015)., Figure S2: Venn diagram (using relevant subsets of the full OTU data set) presenting the sharing of OUTs between sites. CNH—CuoNaHu, HLH—HongLiangHe, GEM—GeErMu., Figure S3: Rarefaction curves of all sequences of different habitats across 3 sites sampled of cold desert, Qinghai Tibet Plateau, China. CNH—CuoNaHu, HLH—HongLiangHe, GEM—GeErMu., Figure S4: Hierarchical cluster analysis of bacterial communities of different habitats across 3 sites sampled of cold desert, Qinghai Tibet Plateau, China: The color intensity in each panel shows the percentage of genera in a sample, referring to the color key at the right top. The top and the left graph show the cluster results of the bacterial community at the genus-level across sites. CNH—CuoNaHu, HLH—HongLiangHe, GEM—GeErMu. Table S1: The information for the research sites., Table S2: Results of the perMANOVA analysis of the Bray-Curtis dissimilarities for bacterial phylum community structure in pH, WC, TN, TOC, EC, TDS and SAL. Df—degrees of freedom; SS—sum of squares; MS —mean sum of squares; Pseudo-F—F value by permutation. *p*-values are based on 9999 permutations., Table S3: Bray-Curtis similarity indices of different habitats across 3 sites of soil sampled of cold desert, Qinghai Tibet Plateau, China., Table S4: Key topological parameters for the co-occurrence network analysis of soil bacterial communities of each site in the cold desert.

## References

[1] Zeng B., Yang T.B. (2009). Natural vegetation responses to warming climates in Qaidam Basin 1982–2003. Int. J. Remote Sens..

[2] Li M., Fang X., Yi C., Gao S., Zhang W., Galy A. (2010). Evaporite minerals and geochemistry of the upper 400 m sediments in a core from the Western Qaidam Basin, Tibet. Quat. Int..

[3] Sul W.J., Asuming-Brempong S., Wang Q., Tourlousse D.M., Penton C.R., Deng J.L.M. (2013). Tropical agricultural land management influences on soil microbial communities through its effect on soil organic carbon. Soil Biol. Biochem..

[4] Xing R., Gao Q.B., Zhang F.Q., Wang J.L., Chen S.L. (2019). Large-scale distribution of bacterial communities in the Qaidam Basin of the Qinghai-Tibet Plateau. Microbiologyopen.

[5] Zhang W., Bahadur A., Zhang G., Zhang B., Wu X., Chen T., Liu G. (2020). Diverse bacterial communities from Qaidam Basin of the Qinghai-Tibet Plateau: Insights into variations in bacterial diversity across different regions. Front. Microbiol..

[6] Makhalanyane T.P., Valverde A., Gunnigle E., Frossard A., Ramond J.B., Cowan D.A. (2015). Microbial ecology of hot desert edaphic systems. FEMS Microbiol. Rev..

[7] Whitman W.B., Coleman D.C., Wiebe W.J. (1998). Prokaryotes: The unseen majority. Proc. Natl. Acad. Sci. USA.

[8] Venter J.C., Remington K., Heidelberg J.F., Halpern A.L., Rusch D., Eisen J.A., Wu D., Paulsen I., Nelson K.E., Nelson W. (2004). Environmental genome shotgun sequencing of the Sargasso Sea. Science.

[9] Shen S. (2017). Community structure and diversity of culturable moderate halophilic bacteria isolated from Qrhan Salt Lake on Qinghai-Tibet Plateau. Acta Ecol. Sin..

[10] Gallardo A., Schlesinger W.H. (1992). Carbon and nitrogen limitations of soil microbial biomass in desert ecosystems. Biogeochemistry.

[11] Tin T., Fleming Z.L., Hughes K.A., Ainley D.G., Convey P., Moreno C.A., Pfeiffer S., Scott J., Snape I. (2008). Impacts of local human activities on the Antarctic environment, Antarctic. Science.

[12] Cederlund H., Stenstrom J. (2004). Microbial biomass and activity on railway track and embankments. Pest Manag. Sci..

[13] Griffiths R.I., Thomson B.C., James P., Bell T., Bailey M., Whiteley A.S. (2011). The bacterial biogeography of British soils. Environ. Microbiol..

[14] Wang J., Zhang T., Li L., Li J., Feng Y., Lu Q. (2017). The patterns and drivers of bacterial and fungal beta-diversity in a typical dryland ecosystem of northwest China. Front. Microbiol..

[15] Whitaker R.J., Grogan D.W., Taylor J.W. (2003). Geographic barriers isolate endemic populations of hyperthermophilic archaea. Science.

[16] Green J.L., Bohannan B.J.M., Whitaker R.J. (2008). Microbial biogeography: From taxonomy to traits. Science.

[17] Fierer N., Jackson R.B. (2006). The diversity and biogeography of soil bacterial communities. Proc. Natl. Acad. Sci. USA.

[18] Stegen J.C., Lin X., Fredrickson J.K., Chen X., Kennedy D.W., Murray C.J., Rockhold M.L., Konopka A. (2013). Quantifying community assembly processes and identifying features that impose them. Int. Soc. Microb. Ecol. J..

[19] Tripathi B.M., Stegen J.C., Kim M., Dong K., Adams J.M., Lee Y.K. (2018). Soil pH mediates the balance between stochastic and deterministic assembly of bacteria. Int. Soc. Microb. Ecol. J..

[20] Zhang B., Wu X., Tai X., Sun L., Wu M., Zhang W., Chen X., Zhang G., Chen T., Liu G. (2019). Variation in actinobacterial community composition and potential function in different soil ecosystems belonging to the arid Heihe River Basin of northwest China. Front. Microbiol..

[21] Miller S.R., Strong A.L., Jones K.L., Ungerer M.C. (2009). Bar-coded pyrosequencing reveals shared bacterial community properties along the temperature gradients of two alkaline hot springs in Yellowstone National Park. Appl. Environ. Microbiol..

[22] Hansel C.M., Fendorf S., Jardine P.M., Francis C.A. (2008). Changes in bacterial and archaeal community structure and functional diversity along a geochemically variable soil profile. Appl. Environ. Microbiol..

[23] Crits-Christoph A., Robinson C.K., Barnum T., Fricke W.F., Davila A.F., Jedynak B., McKay C.P., DiRuggiero J. (2013). Colonization patterns of soil microbial communities in the Atacama Desert. Microbiome.

[24] Valverde A., Makhalanyane T.P., Seely M., Cowan D.A. (2015). Cyanobacteria drive community composition and functionality in rock-soil interface communities. Mol. Ecol..

[25] Kennedy A.C., Stubbs T. (2006). Soil microbial communities as indicators of soil health. Ann. Arid Zone.

[26] Fan M., Li J., Tang Z., Shangguan Z. (2020). Soil bacterial community succession during desertification in a desert steppe ecosystem. Land Degrad. Dev..

[27] Liebner S., Rublack K., Stuehrmann T., Wagner D. (2009). Diversity of aerobic methanotrophic bacteria in a permafrost active layer soil of the Lena Delta, Siberia. Microb. Ecol..

[28] Zhang B., Wu X., Zhang G., Li S., Zhang W., Chen X. (2016). The diversity and biogeography of the communities of actinobacteria in the forelands of glaciers at a continental scale. Environ. Res. Lett..

[29] Wickham H., Chang W. (2015). ggplot2 An Implementation of the Grammar of Graphics. https://www.r-project.org/conferences/useR-2006/Abstracts/Wickham.pdf.

[30] Chao A. (1984). Non-parametric estimation of the classes in a population. Scand. J. Stat..

[31] Oksanen J., Blanchet F.G., Friendly M., Kindt R., Legendre P., McGlinn D.E.A. (2017). Vegan: Community Ecology Package. https://CRAN.Rproject.org/package=vegan.

[32] Clarke K.R., Warwick R.M. (1994). Similarity-based testing for community pattern: The two-way layout with no replication. Mar. Biol..

[33] Henderson-Sellers A., Irannejad P., McGuffie K. (2008). Future desertification and climate change: The need for land-surface system evaluation improvement. Glob. Planet. Chang..

[34] Chen H., Boutros P.C. (2011). VennDiagram: A package for the generation of highly-customizable Venn and Euler diagrams in R. BMC Bioinform..

[35] Griffiths R.I., Thomson B.C., Plassart P., Gweon H.S., Stone D., Creamer R.E., Lemanceau P., Bailey M.J. (2016). Mapping and validating predictions of soil bacterial biodiversity using European and national scale datasets. Appl. Soil Ecol..

[36] Sajjad W., Din G., Rafiq M., Iqbal A., Khan S., Zada S., Ali B., Kang S. (2020). Pigment production by cold-adapted bacteria and fungi: Colorful tale of cryosphere with wide range applications. Extremophiles.

[37] Han Q., Huang J., Long D., Wang X., Liu J. (2017). Diversity and community structure of ectomycorrhizal fungi associated with *Larix chinensis* across the alpine treeline ecotone of Taibai Mountain. Mycorrhiza.

[38] Wang W., Yujun Y., Yang Y., Zhou Y., Zhang S., Wang X., Yang Z. (2020). Impact of anthropogenic activities on the sediment microbial communities of Baiyangdian shallow lake. Int. J. Sediment Res..

[39] Tang J., Tang X., Qin Y., He Q., Yi Y., Ji Z. (2019). Karst rocky desertification progress: Soil calcium as a possible driving force. Sci. Total Environ..

[40] Mapelli F., Marasco R., Fusi M., Scaglia B., Tsiamis G., Rolli E., Fodelianakis S., Bourtzis K., Ventura S., Tambone F. (2018). The stage of soil development modulates rhizosphere effect along a High Arctic desert chronosequence. Int. Soc. Microb. Ecol. J..

[41] Sun Y., Shi Y.L., Wang H., Zhang T., Yu L.Y., Sun H., Zhang Y.Q. (2018). Diversity of bacteria and the characteristics of actinobacteria community structure in Badain Jaran desert and Tengger desert of China. Front. Microbiol..

[42] Van Horn D.J., Van Horn M.L., Barrett J.E., Gooseff M.N., Altrichter A.E., Geyer K.M., Zeglin L.H., Takacs-Vesbach C.D. (2013). Factors controlling soil microbial biomass and bacterial diversity and community composition in a cold desert ecosystem: Role of geographic scale. PLoS ONE.

[43] Quoreshi A.M., Suleiman M.K., Kumar V., Manuvel A.J., Sivadasan M.T., Islam M.A., Khasa D.P. (2019). Untangling the bacterial community composition and structure in selected Kuwait desert soils. Appl. Soil Ecol..

[44] Gupta P., Sangwan N., Lal R., Vakhlu J. (2015). Bacterial diversity of Drass, cold desert in Western Himalaya, and its comparison with Antarctic and Arctic. Arch. Microbiol..

[45] Youssef N.H., Couger M.B., Elshahed M.S. (2010). Fine-scale bacterial beta diversity within a complex ecosystem (Zodletone Spring, OK, USA): The role of the rare biosphere. PLoS ONE.

[46] An S., Couteau C., Luo F., Neveu J., DuBow M.S. (2013). Bacterial diversity of surface sand samples from the Gobi and Taklamaken deserts. Microb. Ecol..

[47] Costello E.K., Schmidt S.K. (2006). Microbial diversity in alpine tundra wet meadow soil: Novel Chloroflexi from a cold, water-saturated environment. Environ. Microbiol..

[48] Wagner D., Kobabe S., Liebner S. (2009). Bacterial community structure and carbon turnover in permafrost-affected soils of the Lena Delta, northeastern Siberia. Can. J. Microbiol..

[49] Pradhan S., Srinivas T.N.R., Pindi P.K., Kishore K.H., Begum Z., Singh P.K., Singh A.K., Pratibha M.S., Yasala A.K., Reddy G.S.N. (2010). Bacterial biodiversity from Roopkund Glacier, Himalayan mountain ranges, India. Extremophiles.

[50] Shivaji S., Pratibha M.S., Sailaja B., Kishore K.H., Singh A.K., Begum Z., Anarasi U., Prabagaran S.R., Reddy G.S.N., Srinivas T.N.R. (2011). Bacterial diversity of soil in the vicinity of Pindari glacier, Himalayan mountain ranges, India, using culturable bacteria and soil 16S rRNA gene clones. Extremophiles.

[51] Scola V., Ramond J.B., Frossard A., Zablocki O., Adriaenssens E.M., Johnson R.M., Seely M., Cowan D.A. (2018). Namib desert soil microbial community diversity, assembly, and function along a natural Xeric Gradient. Microb. Ecol..

[52] Gao S., Liang J., Teng T.M. (2019). Petroleum contamination evaluation and bacterial community distribution in a historic oilfield located in loess plateau in China. Appl. Soil Ecol..

[53] Liu L., Liu Y., Hui R., Xie M. (2017). Recovery of microbial community structure of biological soil crusts in successional stages of Shapotou desert revegetation, northwest China. Soil Biol. Biochem..

[54] Takahashi Y., Matsumoto A., Seino A., Iwai Y., Omura S. (1996). Rare actinomycetes isolated from desert soils. Actinomycetologica.

[55] Sajjad W., Zheng G., Zhang G., Ma X., Xu W., Ali B., Rafiq M. (2018). Diversity of prokaryotic communities indigenous to acid mine drainage and related rocks from Baiyin Open-Pit Copper Mine Stope, China. Geomicrobiol. J..

[56] Stomeo F., Makhalanyane T.P., Valverde A., Pointing S.B., Stevens M.I., Cary C.S., Tuffin M.I., Cowan D.A. (2012). Abiotic factors influence microbial diversity in permanently cold soil horizons of a maritime-associated Antarctic Dry Valley. FEMS Microbiol. Ecol..

[57] Sher Y., Zaady E., Nejidat A. (2013). Spatial and temporal diversity and abundance of ammonia oxidizers in semi-arid and arid soils: Indications for a differential seasonal effect on archaeal and bacterial ammonia oxidizers. FEMS Microbiol. Ecol..

[58] Andrew D.R., Fitak R.R., Munguia-Vega A., Racolta A., Martinson V.G., Dontsova K. (2012). Abiotic factors shape microbial diversity in Sonoran Desert soils. Appl. Environ. Microbiol..

[59] Wang X., Van Nostrand J.D., Deng Y., Lu X., Wang C., Zhou J., Han X. (2015). Scale-dependent effects of climate and geographic distance on bacterial diversity patterns across northern China’s grasslands. FEMS Microbiol. Ecol..

[60] Ganzert L., Lipski A., Hubberten H.W., Wagner D. (2011). The impact of different soil parameters on the community structure of dominant bacteria from nine different soils located on Livingston Island, South Shetland Archipelago, Antarctica. FEMS Microbiol. Ecol..

[61] Arocha-Garza H.F., Canales-Del Castillo R., Eguiarte L.E., Souza V., De la Torre-Zavala S. (2017). High diversity and suggested endemicity of culturable Actinobacteria in an extremely oligotrophic desert oasis. PeerJ.

[62] Varin T., Lovejoy C., Jungblut A.D., Vincent W.F., Corbeil J. (2012). Metagenomic analysis of stress genes in microbial mat communities from Antarctica and the High Arctic. Appl. Environ. Microbiol..

[63] Inskeep W.P., Jay Z.J., Tringe S.G., Herrgard M.J., Rusch D.B., Committee M. (2013). Working Group, The YNP Metagenome project: Environmental parameters responsible for microbial distribution in the yellowstone geothermal ecosystem. Front. Microbiol..

[64] Tecon R., Or D. (2017). Biophysical processes supporting the diversity of microbial life in soil. FEMS Microbiol. Rev..

[65] Chu H., Sun H., Tripathi B.M., Adams J.M., Huang R., Zhang Y., Shi Y. (2016). Bacterial community dissimilarity between the surface and subsurface soils equals horizontal differences over several kilometers in the western Tibetan Plateau. Environ. Microbiol..

[66] Jiang Q., Yang X. (2019). Sedimentological and geochemical composition of aeolian sediments in the Taklamakan desert: Implications for provenance and sediment supply mechanisms. J. Geophys. Res..

[67] Ronca S., Ramond J.B., Jones B.E., Seely M., Cowan D.A. (2015). Namib Desert dune/interdune transects exhibit habitat-specific edaphic bacterial communities. Front. Microbiol..

[68] Waheed H., Hashmi I., Naveed A.K., Khan S.J. (2013). Molecular detection of microbial community in a nitrifying–denitrifying activated sludge system. Int. Biodeterior. Biodegrad..

[69] Li J., Liu W., Luo L., Dong D., Liu T., Zhang T., Lu C., Liu D., Zhang D., Wu H. (2015). Expression of *Paenibacillus polymyxa* β-1,3-1,4-glucanase in Streptomyces lydicus A01 improves its biocontrol effect against Botrytis cinerea. Biolog. Control..

[70] Fierer N., Leff J.W., Adams B.J., Nielsen U.N., Bates S.T., Lauber C.L., Owens S., Gilbert J.A., Wall D.H., Caporaso J.G. (2012). Cross-biome metagenomic analyses of soil microbial communities and their functional attributes. Proc. Natl. Acad. Sci. USA.

[71] De Cáceres M., Legendre P., Moretti M. (2010). Improving indicator species analysis by combining groups of sites. Oikos.

[72] Tapia-Torres Y., López-Lozano N.E., Souza V., García-Oliva F. (2015). Vegetation-soil system controls soil mechanisms for nitrogen transformations in an oligotrophic Mexican desert. J. Arid Environ..

[73] Zhang B., Kong W., Wu N., Zhang Y. (2016). Bacterial diversity and community along the succession of biological soil crusts in the Gurbantunggut Desert, Northern China. J. Basic Microbiol..

[74] Long H., Kucukyildirim S., Sung W., Williams E., Lee H., Ackerman M., Doak T.G., Tang H., Lynch M. (2015). Background mutational features of the radiation-resistant bacterium Deinococcus radiodurans. Mol. Biol. Evol..

[75] Sajjad W., Ali B., Bahadur A., Ghimire P.S., Kang S. (2020). Bacterial diversity and communities structural dynamics in soil and meltwater runoff at the frontier of Baishui Glacier No.1, China. Microb. Ecol..

