# Supplementary material for: Bacterial Diversity and Community Composition Distribution in Cold-Desert Habitats of Qinghai–Tibet Plateau, China
Source: Microorganisms. 2021 Jan 27;9(2):262. doi: 10.3390/microorganisms9020262 (PMC7911287; doi:10.3390/microorganisms9020262)
Supplement: Supplementary file 1 [file microorganisms-09-00262-s001.pdf]

Table S1 The information for the research sites

Site	Abbr.	Long.	Lat.	El. (m)	Distance (m)	Vegetation
GeErMu	G-GH	94°22′E	35°49′E	3940	40	70-80%
	G-GF	94°22′E	35°49′E	3940	20	30-50%
	G-DL	94°22′E	35°49′E	3940	10	Non
	G-HD	94°22′E	35°49′E	3940	0[a]	Non
	G-SD	94°22′E	35°49′E	3940	200	Non
HongLiangHe	H-GH	93°00′N	35°02′E	4633	40	70-80%
	H-GF	93°00′N	35°02′E	4633	20	30-50%
	H-DL	93°00′N	35°02′E	4633	10	Non
	H-HD	93°00′N	35°02′E	4633	0[a]	Non
	H-SD	93°00′N	35°02′E	4633	200	Non
CuoNaHu	C-GH	91°35′N	32°08′E	4624	40	70-80%
	C-GF	91°35′N	32°08′E	4624	20	30-50%
	C-DL	91°35′N	32°08′E	4624	10	Non
	C-HD	91°35′N	32°08′E	4624	0[a]	Non
	C-SD	91°35′N	32°08′E	4624	200	Non

[a] Distance from the railway track

Table S2 Results of the perMANOVA analysis of the Bray-Curtis dissimilarities for bacterial phylum community structure in pH, WC, TN, TOC, EC, TDS and SAL. Df = degrees of freedom; SS = sum of squares; MS = mean sum of squares; Pseudo-F = F value by permutation. *P*-values are based on 9999 permutations.

	**Df**	**SS**	**MS.**	***F***	R^2	***P***
**pH**	1	0.0177	0.0177	0.6812	0.0302	< 0.05
**WC**	1	0.0556	0.0556	2.1408	0.0949	< 0.05
**TN**	1	0.1327	0.1327	5.1069	0.2266	0.005 **
**TOC**	1	0.0339	0.0339	1.3047	0.0579	< 0.05
**EC**	1	0.1370	0.1370	5.2743	0.2340	0.006 **
**TDS**	1	0.01573	0.0157	0.6053	0.0268	< 0.05
**SAL**	1	0.0110	0.0110	0.4236	0.0188	< 0.05

Significant codes: 0 '***'; 0.001'**'; 0.01 '*'; 0.05 '.'

Table S3 Bray-Curtis similarity indices of different habitats across 3 sites of soil sampled of cold desert, Qinghai Tibet Plateau, China.

	G-GH	G-GF	G-DL	G-HD	G-SD	H-GH	H-GF	H-DL	H-HD	H-SD	C-GH	C-GF	C-DL	C-HD	C-SD
G-GH	1														
G-GF	0.457	1													
G-DL	0.314	0.253	1												
G-HD	0.385	0.292	0.479	1											
G-SD	0.239	0.266	0.224	0.189	1										
H-GH	0.495	0.354	0.187	0.204	0.226	1									
H-GF	0.444	0.450	0.301	0.322	0.236	0.441	1								
H-DL	0.345	0.483	0.283	0.258	0.435	0.334	0.449	1							
H-HD	0.329	0.223	0.233	0.192	0.201	0.311	0.295	0.285	1						
H-SD	0.442	0.402	0.310	0.294	0.289	0.457	0.479	0.465	0.459	1					
C-GH	0.480	0.424	0.297	0.307	0.255	0.524	0.616	0.421	0.326	0.477	1				
C-GF	0.398	0.374	0.398	0.514	0.238	0.279	0.507	0.359	0.244	0.378	0.501	1			
C-DL	0.405	0.465	0.328	0.337	0.267	0.365	0.508	0.457	0.299	0.482	0.560	0.464	1		
C-HD	0.380	0.409	0.263	0.250	0.300	0.399	0.445	0.462	0.356	0.501	0.482	0.351	0.538	1	
C-SD	0.444	0.417	0.293	0.286	0.268	0.416	0.507	0.401	0.361	0.514	0.573	0.411	0.601	0.571	1

Table S4 Key topological parameters for the co-occurrence network analysis of soil bacterial communities of each site in the cold desert.

Network attributes	GEM	HLH	CNH
**Nodes**	94	101	93
**Edges**	162	157	102
**Int. Positives**	22.84%	44.49%	38.24%
**Int. Negatives**	77.16%	55.51%	61.78%
**Average Degree**	3.45	3.11	2.19
**Modularity**	0.34	0.43	0.79
**Avg. weighted degree**	1.72	1.55	1.09

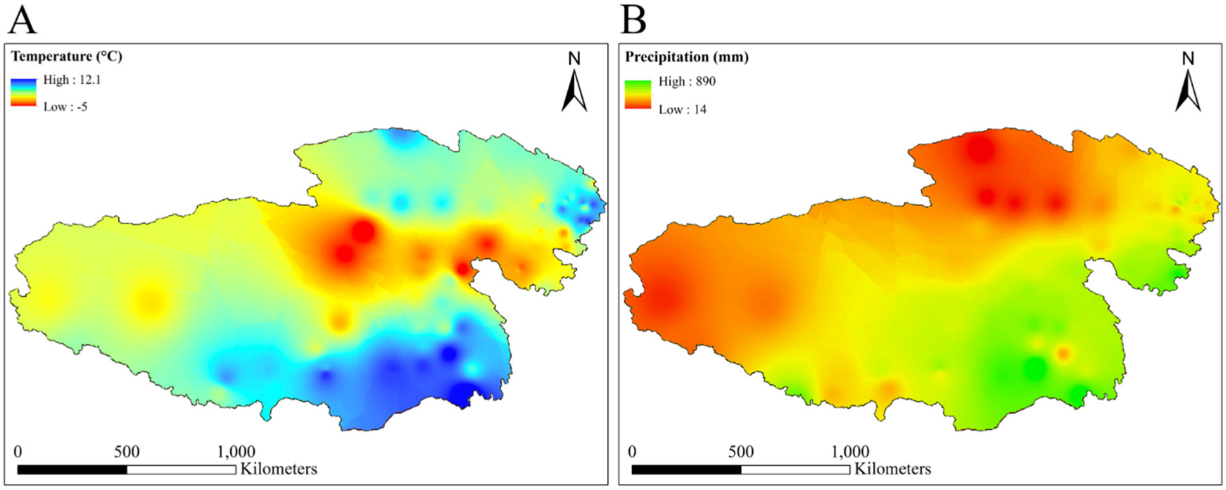

Figure S1. (A) Temperature data obtained from ordinary Inverse distance weighting (IDW) interpolation based on the 30-year average annual records (1981 to 2015). (B) Precipitation map obtained from ordinary Inverse distance weighting (IDW) interpolation based on the 30-year average annual records (1981 to 2015).

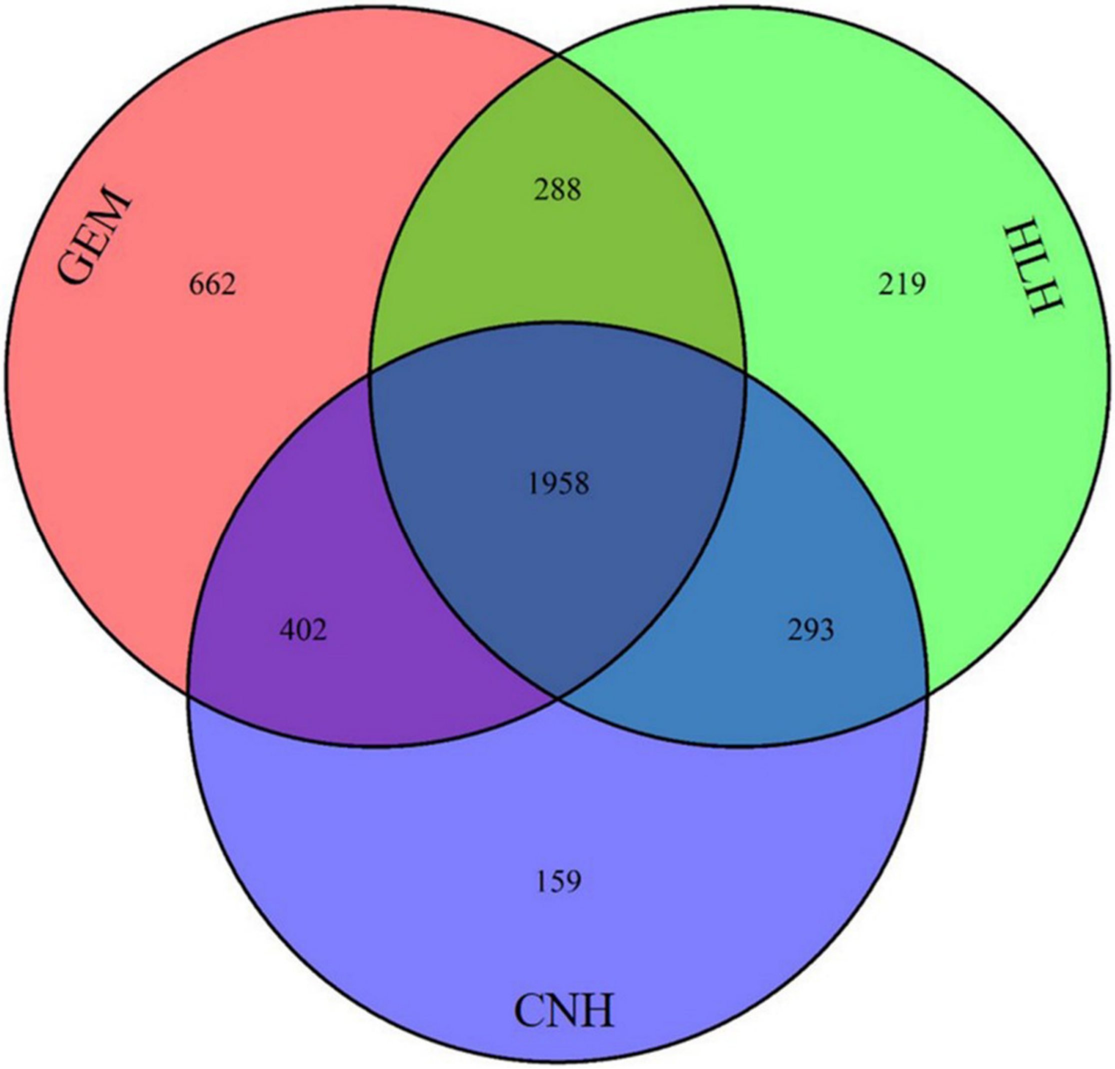

Figure S2. Venn diagram (using relevant subsets of the full OTU data set) presenting the sharing of OUTs between sites. CNH = CuoNaHu, HLH = HongLiangHe, GEM = GeErMu.

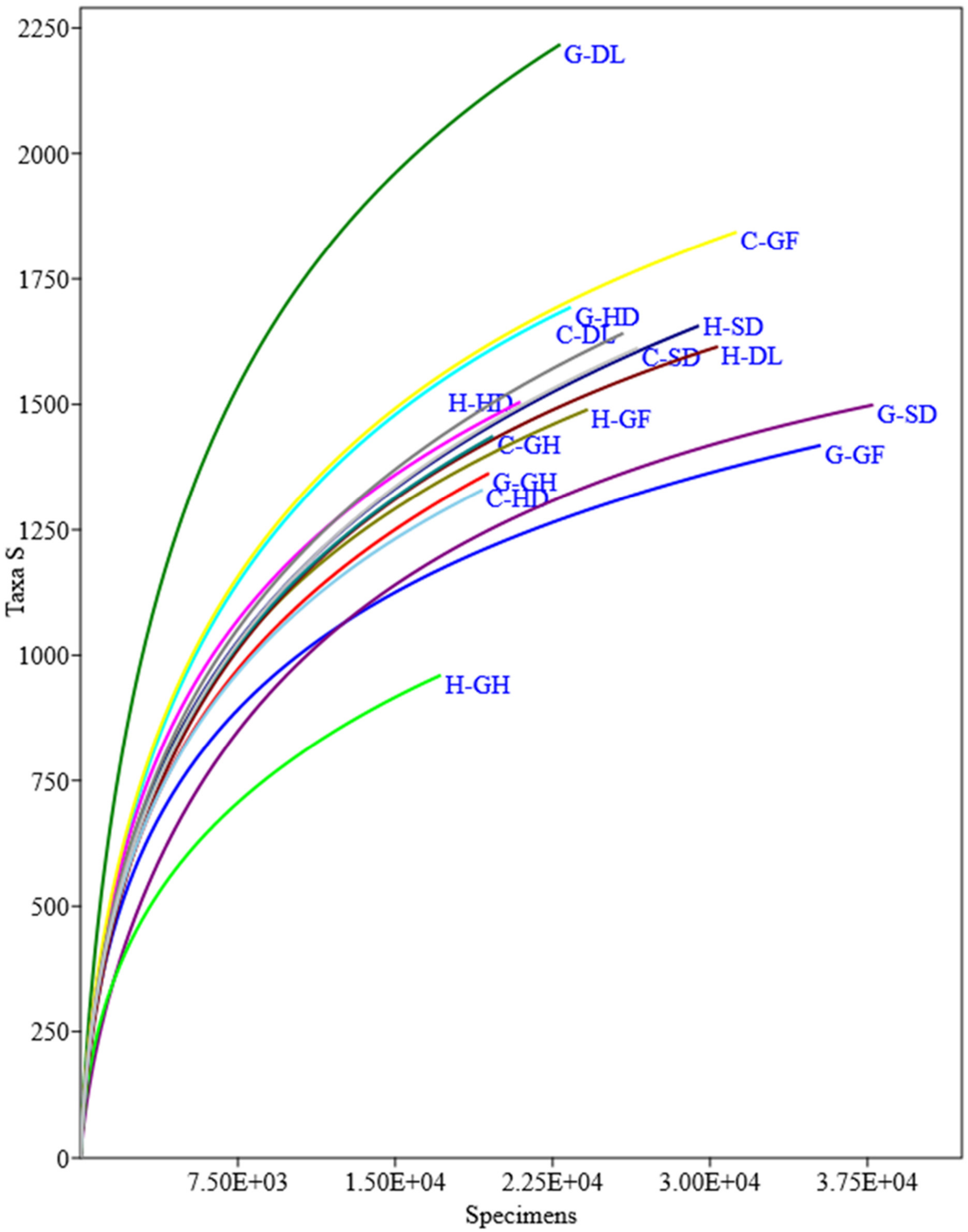

Figure S3. Rarefaction curves of all sequences of different habitats across 3 sites sampled of cold desert, Qinghai Tibet Plateau, China. CNH = CuoNaHu, HLH = HongLiangHe, GEM = GeErMu.

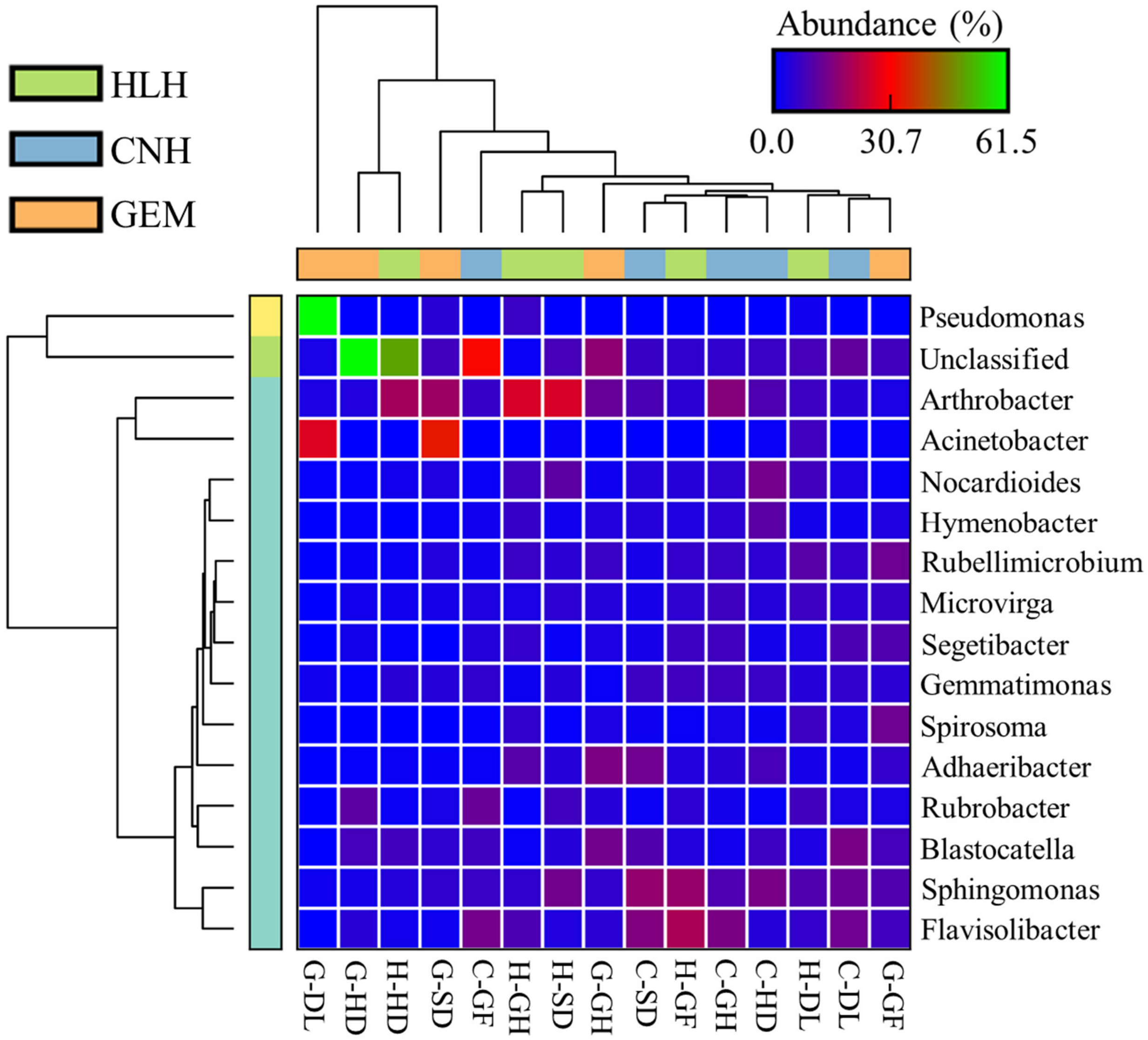

Figure S4. Hierarchical cluster analysis of bacterial communities of different habitats across 3 sites sampled of cold desert, Qinghai Tibet Plateau, China: The color intensity in each panel shows the percentage of genera in a sample, referring to the color key at the right top. The top and the left graph show the cluster results of the bacterial community at the genus-level across sites. CNH = CuoNaHu, HLH = HongLiangHe, GEM = GeErMu.
